# Oligodendroglioma: A Review of Management and Pathways

**DOI:** 10.3389/fnmol.2021.722396

**Published:** 2021-10-05

**Authors:** Maroun Bou Zerdan, Hazem I. Assi

**Affiliations:** Division of Hematology and Oncology, Department of Internal Medicine, Naef K. Basile Cancer Institute, American University of Beirut Medical Center, Beirut, Lebanon

**Keywords:** anaplastic oligodendroglioma, PCV, IDH mutation, procarbazine, lomustine, vincristine, temozolomide

## Abstract

Anaplastic oligodendrogliomas are a type of glioma that occurs primarily in adults but are also found in children. These tumors are genetically defined according to the mutations they harbor. Grade II and grade III tumors can be differentiated most of the times by the presence of anaplastic features. The earliest regimen used for the treatment of these tumors was procarbazine, lomustine, and vincristine. The treatment modalities have shifted over time, and recent studies are considering immunotherapy as an option as well. This review assesses the latest management modalities along with the pathways involved in the pathogenesis of this malignancies.

## Introduction

Oligodendroglial tumors are rare tumors that constitute part of the neuro epithelial tumors of the central nervous system. Accounting to up to 5% of all neuroepithelial tumors (Ostrom et al., 2017), oligodendroglial tumors have an incidence rate of around 1,000 new cases per year in the United States. Oligodendroglial tumors can be divided into two groups based on the classification of the world health organization (WHO): grade II oligodendroglioma and grade III (anaplastic) oligodendroglioma. Most commonly occurring between 25 and 45 years of age, grade III oligodendrogliomas tend to present 10 years later than grade II tumors and can rarely develop in younger and older populations. Oligodendroglioma is genetically defined as a tumor confirmed to harbor either an IDH1 or IDH2 mutation along with co-deletion of chromosome arms 1p and 19q. Histologically, oligodendroglial tumors show sheets of isomorphic round nuclei with a clear cytoplasm—the classic “fried egg” appearance. Grade III oligodendroglioma show a worse prognosis than grade II tumors due to the presence of anaplastic features such as nuclear atypia, necrosis, microvascular proliferation, high cell density and number of mitotic figures. It is believed that anaplastic oligodendroglioma (AO) can progress from a lower grade oligodendroglioma after the acquisition of specific genetic alterations (Youssef and Miller, 2020). However, a clear distinction of both grades is not always possible. Increasing interest has been projected toward favorable molecular markers which oligodendrogliomas harbor. In this review article, we describe the clinical management of AO and summarize the different molecular pathways that drive the development, maintenance, and treatment response of these tumors.

## Current Management Guidelines

To establish the diagnosis of AO, a pathological sample is crucial. Hence, surgeons should biopsy patients suspected to have AO and attempt tumor resection, as with all other high-grade gliomas. In a study by Shin et al. (2020), gross tumor resection (GTR) was done in 43 of 88 patients. Upon multivariate analysis, median progression free survival (PFS) was 41.1 vs. 23.9 months along with a hazard ratio (HR) of 0.58 with a 95% CI 0.35–0.97 (*p* = 0.038) compared to patients who had no GTR (Shin et al., 2020). However, upon multivariate analysis there was no significant difference in overall survival (OS). Similarly in a retrospective study by Fujii et al. (2017) patients with anaplastic astrocytoma or anaplastic oligoastrocytoma but not AO had a significant survival advantage when resection of at least 53% of the preoperative T2-weighted high-signal intensity volume was done. Alattar et al. (2018) conducted a Surveillance, Epidemiology, and End Results (SEER)-based analysis in 2017 and showed that GTR was not associated with improved survival in patients with WHO grade II and grade III oligodendrogliomas compared to patients with anaplastic astrocytomas and glioblastomas. This was attributed to the sensitivity of oligodendrogliomas to chemotherapy compared to astrocytomas (Alattar et al., 2018).

Although surgery can help relieve symptoms by decreasing the mass effect of the tumor, the tumor’s predilection to the frontal lobe hinders its maximal resection. This comes with a risk of sacrificing important brain centers and hence compromising functionality and quality of life. Retrospective studies have revealed that the post-operative seizure-free rate is 67–80% (Luyken et al., 2003; Zaatreh et al., 2003; Benifla et al., 2006; Chang et al., 2008; Englot et al., 2011). Despite utilizing a multimodal approach in nearly all patients, refractory seizures can still be seen in patients suffering from epilepsy in 50% of the cases before the initial surgery and 15–40% of cases following surgery and anticonvulsant therapy (Smits and Duffau, 2011; You et al., 2011; Calatozzolo et al., 2012). Two plausible hypotheses to explain treatment resistance in oligodendrogliomas exist. The first is the presence of alterations in drug targets affecting antiepileptic drugs’ binding. The second is diminished intracellular drug transport through the overexpression of ATP-binding cassette transporter proteins such as P-gp (MDR1), MRP1, and MRP5 (Calatozzolo et al., 2012; Alms et al., 2014).

Postoperative radiotherapy (XRT) to a total dose of roughly 60 Gy over 30 fractions is recommended (Blakeley and Grossman, 2008). Although one survey showed that 34% of neuro-oncologists suggested delaying XRT in patients with 1p19 co-deletions (Abrey et al., 2007), clinical trials addressing the efficacy of delayed XRT in this subset of patients are needed.

The earliest reported results of the chemotherapy regimens, procarbazine, lomustine (CCNU), and vincristine (PCV), in AO were reported by Cairncross et al. (1994) and showed that the median time to progression for patients was at least 25.2 months for complete responders, 14.2 months for partial responders and 6.8 months for stable patients. Afterward in 2001, Chinot et al. (2001) reported that 16.7% of patients experienced a complete response and 27.1% experienced a partial response when receiving temozolomide (TMZ) after previous PCV. The radiation Therapy Oncology Group (RTOG) also explored the use of pre-irradiation TMZ followed by concurrent TMZ and radiotherapy in a phase 2 study (RTOGBR013) (Vogelbaum et al., 2009).

Accordingly, the treatment approach is tailored according to the presence of 1p19q co-deletion, which characterizes oligodendrogliomas. Patients harboring co-deleted tumors can receive either PCV or TMZ. The European Organization for Research and Treatment of Cancer study 26951 (EORTC26951) and RTOG9402 showed an increase in OS and PFS when PCV is added to radiotherapy (RT) in patients with 1p19q co-deleted oligodendrogliomas (Cairncross et al., 2013; van den Bent et al., 2013a). With almost 12 years of follow-up, patients harboring tumors with 1p19q co-deletions showed an improved survival when treated with PCV and RT as compared to RT alone (EORTC26951: 157 vs. 50 months; RTOG9402: 14.7 vs. 7.3 years). Moreover, treatment of these patients with PCV demonstrated an improved OS in both groups when compared to RT alone. In patients with astrocytic tumors, only PFS was prolonged in patients treated with XRT who received up-front PCV vs. PCV at the time of recurrence (Pan-Weisz, 2019; Tork and Atkinson, 2020). Consequently, and in terms of improvement in quality of life (QOL), the EORTC study showed no difference between the two groups, and PCV toxicity contributed to a decreased QOL for a prolonged period.

The response of tumors harboring IDH mutations to PCV therapy has also been described in a subset analysis and follow-up study of RTOG9402 trial. As expected, patients with an IDH mutation and 1p19q co-deletion showed significant benefit in OS. While IDH-WT tumors retained a poor prognosis and showed no benefit from PCV treatment, improved OS was seen in IDH mutant non-co-deleted tumors, and astrocytic tumors when treated with PCV plus RT.

A subset analysis of patients with other methylation profiles, such as CpGisland hypermethylated phenotype (CIMP) and MGMT promoter methylation (MGMT-STP27) status, was also conducted by van den Bent et al. (2013b). It was found that CIMP + or MGMT-STP27 methylated tumors had a superior OS 1.05 vs. 6.46 years and 1.06 vs. 3.8 years (both *P* < 0.0001) for CIMP and MGMT-STP27 status, respectively. CIMP + and MGMT-STP27 methylated tumors had a clear benefit from adjuvant PCV; the median OS in the RT and RT-PCV arms was 3.27 vs. 9.51 years (*P* < 0.0033), respectively for CIMP + tumors and 1.98 vs. 8.65 years (*P* < 0.0001) for MGMT-STP27 methylated tumors (van den Bent et al., 2013b). There was however no such benefit for CIMP- or for MGMT-STP27 unmethylated tumors.

Adjuvant TMZ has also been shown to be effective with better tolerability and less toxicity (van den Bent et al., 2003; Brandes et al., 2006). A randomized clinical trial is currently in progress to compare the efficacy of PCV or TMZ when combined with RT in 1p19q co-deleted tumors (CODEL: NCT00887146). Preliminary results are mentioned toward the end of the manuscript. For patients with astrocytic tumors, EORTC26951 and RTOG9402 did not show any benefit of PCV with RT. A trial of adjuvant TMZ with RT in patients harboring this tumor subtype showed a significantly improved PFS and OS (van den Bent et al., 2017). While increasing the risk of toxicity, concurrent TMZ is currently being assessed in comparison to adjuvant treatment in astrocytic tumors (van den Bent et al., 2017). The interim report from the RTOG0131 trial suggests that combination therapy with TMZ and XRT is well tolerated in patients with AO being treated with neoadjuvant TMZ for 6 months, followed by TMZ and concurrent XRT (Tork and Atkinson, 2020). Apart from RTOG9402 and EORTC26951, Wick et al., 2016 conducted NOA-4, a randomized phase 3 trial of sequential RT followed by chemotherapy against anaplastic glioma with PCV or TMZ (Vogelbaum et al., 2009). In this trial, MGMT hypermethylation was associated with prolonged PFS in both arms (Wick et al., 2009; Tork and Atkinson, 2020).

All in all, patients with 1p19q co-deleted tumors should be treated with RT and adjuvant PCV while those lacking this co-deletion should receive adjuvant TMZ. PCV and TMZ are also used in cases of recurrence but result in lower response rates and disease-free survival. Other agents have also been investigated for recurring disease including paclitaxel, irinotecan, carboplatin, etoposide, and cisplatin (Poisson et al., 1991; Yung et al., 1991; Warnick et al., 1994; Chamberlain and Kormanik, 1995, 1999; Fulton et al., 1996; Macdonald et al., 1996; Friedman et al., 1999; Chang et al., 2001; Cloughesy et al., 2003; Batchelor et al., 2004; Ascierto et al., 2016). However, no results have proven enough benefit for treating patients with recurrent AO. Table 1 outlines some information related to the major drugs used in treatment.

**TABLE 1:** Major drugs utilized in the treatment of AO.

	Procarbazine	Lomustine (CCNU)	Vincristine	Temozolomide (TMZ)
Standard dosage	60 mg/m^2^ daily on days 8–21.	110 mg/m^2^ on day 1; repeat every 8 weeks.	1.4 mg/m^2^ (IV) on days 8 and 29.	75 mg/m^2^ daily for about 42 consecutive days when given with XRT^a^. The adjuvant dosage is 150–200 mg/m^2^ for 5 of every 28 days.
Contraindications	Hypersensitivity to drug or components, myelosuppression.	Hypersensitivity to nitrosoureas, inadequate bone marrow function, history of pulmonary fibrosis.	Hypersensitivity to the drug or components, intrathecal administration, history of familial or other advanced PN^b^.	Hypersensitivity to TMZ or dacarbazine; pregnancy, nursing.
Main drug interactions	Disulfiram-like interaction with alcohol. Tricyclic antidepressants and sympathomimetics may induce hypertensive crisis. Rare cases of encephalopathy.	Phenobarbital may decrease efficacy.	Cytochrome P450 inhibitors may increase vincristine serum concentrations.	Administration of valproic acid decreases oral clearance of TMZ by about 5%;
Main side effects	Encephalopathy, nausea, vomiting, myelosuppression, photosensitivity.	Nausea, vomiting, myelosuppression, pulmonary fibrosis.	Neuropathy (peripheral or autonomic) is the most common, dose-limiting toxicity.	Nausea, vomiting, myelosuppression (thrombocytopenia).

*The standard dosage of PCV combination is every 6–8 weeks in the following order: CCNU (110 mg/m^2^) on day 1; intravenous vincristine (1.4 mg/m^2^) on days 8 and 29; oral procarbazine (60 mg/m^2^) daily on days 8–21.*

*^a^XRT, Radiotherapy.*

*^b^PN, peripheral neuropathy.*

Recently, immunotherapy has been explored as a potential treatment modality. Elens et al. (2012) reported the survival benefit of immunotherapy in patients with relapsed AO enrolled in the HGG-IMMUNO-2003 trial. The PFS and OS were 3.4 and 18.8 months, respectively. In a recent case report by Yu et al. (2021) a patient who had multiple tumor recurrences, following several regimens was started eventually on nivolumab. On magnetic resonance imaging, he was considered to have disease progression. Upon surgical debulking and pathological diagnosis, he was found to have recurrent diseases. However, tumor samples collected from enhancing and non-enhancing areas for a scRNAseq analysis revealed an abundance of immune cells. Infiltration of these cells might have been perceived as the increased mass on MRI. The patient sustained a disease-free response to nivolumab at least 12 months after surgery. This highlights the importance in incorporating novel techniques to better understand the tumor microenvironment (Yu et al., 2021).

## Pathways in Anaplastic Oligodendroglioma

### Isocitrate Dehydrogenase 1/2 Mutation (IDH 1/2)

The main function of the IDH1 and IDH2 enzymes is the oxidative decarboxylation of isocitrate to alpha-ketoglutarate. This reaction promotes the formation of NADPH, the reduced form of NADP+, which helps in protecting the cell from oxidative radicals that can damage DNA (Soffietti et al., 1998; van den Bent et al., 1998). In the cytosol, the product of the reaction catalyzed by IDH1, alpha-KG, has been reported to be involved in multiple cellular pathways including hypoxia sensing, lipogenesis and epigenetic modification through its action on alpha-KG dependent dioxygenases such as TET and JmjC and other enzymes (Mason et al., 1996; Buckner et al., 2003; Abrey et al., 2006; Taliansky-Aronov et al., 2006). The role of IDH2, on the other hand, is limited to the mitochondria where it catalyzes the same reaction as part of the tricarboxylic acid cycle (TCA).

IDH mutations identified in gliomas tend to occur at the active site of the enzyme at arginine 132 and 172 in IDH1 and IDH2, respectively. IDH mutations can dominantly inhibit WT-IDH when heterozygous through the formation of enzymatically inactive heterodimers (Zhao et al., 2009). It was shown by Uhm (2010) that IDH mutations lead to the acquisition of a new enzymatic function that catalyzes the formation of D-2HG from alpha-KG. 2-HG can inhibit alpha-KG dependent dioxygenases and cause epigenetic alterations (Xu et al., 2011). It can also stimulate the activity of EGLN leading to decreased HIF levels. This in turn allows tumor proliferation in low oxygen conditions (Zhao et al., 2009; Koivunen et al., 2012). It has also been reported that 2-HG can inhibit p53 via microRNA activated by HIF-2α, driving tumorigenesis (Jiang et al., 2018). As a result of the disruption of IDH’s enzymatic function, 2-HG tilts off the NADP/NADPH balance thereby increasing the production of ROS and leading to DNA damage and tumor formation (Latini et al., 2003; Rinaldi et al., 2016). JmjC demethylases are one of the many dioxygenases regulated by α-KG and inhibited by 2-HG. They are responsible for histone methylation on lysine residues. It has been observed that in IDH-mutant cell lines, repressive histone methylation precedes global DNA hypermethylation. This in turn provides evidence that IDH mutations could allow cells to remain in a vulnerable state, and prone to additional DNA alterations. Mutant IDH1 has also been shown to inhibit the ALkB family DNA repair enzymes further contributing to erroneous DNA replication (Wang et al., 2015; Rinaldi et al., 2016).

High mutant allele fractions have been found in patient samples at diagnosis and recurrence in tumor evolution studies. IDH1 mutations seem to be at the core of this tumorigenesis (Johnson et al., 2014). IDH mutated enzymes can promote proliferation and colony formation through its end metabolite 2-HG (Koivunen et al., 2012; Bittinger et al., 2013). Turcan et al. (2012) showed that an IDH1 mutation can induce a methylation profile known as the G-CIMP signature, which is a glioma specific methylation pattern at CpG islands. Interestingly, an *in vitro* treatment of cells with D-2HG also induced a similar methylation pattern (Lu et al., 2012) which further supports the vital role of this metabolite in epigenetic alteration and tumor formation. Additionally, hypermethylation caused by IDH1 mutations was shown to occur at CTCF-binding sites that normally insulate and prevent the interaction between different parts of the genome (Flavahan et al., 2016). Methylation of these sites promotes the interaction of enhancers with new genes (Flavahan et al., 2016).

IDH mutations have also been implicated in the regulation of the recruitment of inflammatory cells to tumor sites, specifically through D-2HG. Evidence from *in vivo* models have demonstrated reduced levels of STAT1 and CXCL10 in IDH-mutant gliomas. Infiltration of immune cells, specifically T cells, were also reduced in these tumors (Amankulor et al., 2017; Kohanbash et al., 2017). Additionally, the mTOR pathway has been identified at a potential target for treatment due its activation in IDH-mutant gliomas. This occurs via 2-HG’s inhibition of KDM4A, an α-KG dependent deoxygenase, and destabilization of DEPTOR, a negative regulator of mTORC1/2, resulting in mTOR pathway activation (Carbonneau et al., 2016). This activation is of special interest since it has been shown that mTOR and its downstream effectors are implicated in tumorigeneses in brain malignancies (Fan and Weiss, 2010; Ryskalin et al., 2017).

The RTOG 9802 trial, which included non-molecularly stratified patients harboring grade II gliomas, demonstrated a 5.5-year survival benefit of PCV administration (Shaw et al., 2008). Results of this trial raise the possibility that the chemosensitivity seen in these tumors might be due to the IDH mutation that is common to both oligodendroglial and low-grade astrocytic gliomas. Upon reanalysis of RTOG 9802 after molecular classification, AO patients with IDH-mutated tumors actually showed a survival benefit when treated with PCV chemotherapy (Cairncross et al., 2014). However, analysis of other trials such as the EORTC 26951 did not reveal a correlation between IDH mutations and survival in patients with astrocytic tumors (grade II) (van den Bent et al., 2010, 2013a). Appropriate design of future clinical trials can help in determining better correlations with molecular subclasses.

### Chromosome 1p19q Co-deletion: Role of CIC, FUBP1, and NOTCH1 in Promoting Carcinogenesis

The unbalanced translocation of the centromeric regions of chromosomes 1p and 19q attribute to the loss of the whole arm on both chromosomes. This co-deletion, along with the IDH mutation, enables a tumor to be classified as an oligodendroglioma according to the WHO 2016 criteria (Louis et al., 2016). Patients with co-deleted tumors demonstrate favorable prognoses (Smith et al., 2000a; Ino et al., 2001; Cairncross et al., 2006; Kaloshi et al., 2007; Cairncross et al., 2013). The mechanism by which this co-deletion leads to chemosensitivity remains unclear and data showing the implication of other genes in this chemosensitivity is emerging.

Following the stratification of AO according to 1p/19q co-deletion status, an in-depth genetic analysis of 1p/19q co-deleted tumors revealed inactivating mutations affecting the *FUBP1* gene on chromosome 1p and the *CIC* gene on chromosome 19 (Bettegowda, 2000; Sahm et al., 2012; Yip et al., 2012). CIC normally functions as a reversible repressor by binding to the DNA regulatory elements downstream of growth factor signaling pathways (Ajuria et al., 2011). Acting as a tumor-suppressor gene, missense mutations affecting CIC are mostly found within the DNA-binding domain thereby inhibiting its binding to regulatory elements. In a population of patients with oligodendroglial tumors, four cases exhibited absent CIC expression with no detectable mutations, suggesting that alterations affecting CIC could occur through other unidentified mechanisms (Chan et al., 2014). Another DNA-binding protein found mutated in AO is FUBP1. The Far Upstream Element (FUSE) Binding Protein 1 (FUBP1) is known to regulate several cell cycle regulators such as MYC and p21. While often found upregulated in many tumors, FUBP1 acts as a tumor suppressor gene due to its inactivating mutations reported in around 15% of oligodendroglial tumors (Baumgarten et al., 2014). As for the clinical relevance of these molecular markers, inactivating mutations affecting FUBP1 have correlated with a shorter time to recurrence and CIC mutations have been associated with worse prognosis, especially in those patients with 1p/19q co-deleted oligodendrogliomas (Chan et al., 2014; Michaud et al., 2018). Nevertheless, further studies are needed to elucidate the role of CIC/FUBP1 alterations in the pathogenesis of AO and oligodendrogliomas, in general.

### CDKN2A Homozygous Deletion/9p LOH: Role in Progression to Anaplastic Oligodendroglioma

In addition to the aforementioned pathways, homozygous and the less common hemizygous losses of 9p21 have been reported with high frequencies in gliomas, and up to 55% in AO (Maruno et al., 1996; Perry et al., 1999; Rasheed et al., 2002; Ohgaki and Kleihues, 2009; Michaud et al., 2018). These alterations have correlated with a shorter event free survival (EFS; 29 vs. 53 months, *p* < 0.0001) and OS (48 vs. 83 months, *p* < 0.0001). At the molecular level, 9p losses result in the loss of the cyclin-dependent kinase inhibitor *CDKN2A* gene, which normally inhibits cellular division. CDKN2A inhibits the interaction between the cyclin dependent-kinases CDK4 or CDK6 and D-type cyclins, preventing both the phosphorylation of the retinoblastoma (RB1) protein and the release of the elongation factor (EF2) (Weinberg, 1995; Sherr and Roberts, 1999). Hence, cellular proliferation and dysregulation of pro-apoptotic pathways ensues (Ruas and Peters, 1998). Although 9p losses can be found in many gliomas, they more commonly occur in higher grade tumors (grades 3 and 4), which make the *CDKN2A* gene or p16 protein (*CDKN2A* product) potential players involved in the malignant progression and anaplastic transformation of low-grade gliomas into higher grades (He et al., 1995; Ueki et al., 1996; Watanabe et al., 2001). Interestingly, some tumors exhibited p16 hyperexpression without any chromosome 9p alterations and this was associated with a shorter EFS and OS. Cyclin D1 expression was also significantly higher in AO and was associated with a shorter EFS (Michaud et al., 2018).

### TCF12 Transcription Factor Mutations

TCF12 protein is a transcription factor and member of the basic helix-loop-helix (bHLH) E-protein family. Through the formation of homo- and hetero-dimers with other bHLH transcription factors, TCF12 modulates the transcription of specific genes that are intrinsic to the oligodendrocyte lineage (Fu et al., 2009) and are involved in neural development (Uittenbogaard and Chiaramello, 2002). Two main alterations affecting the TCF12 protein have been reported in AO: absence of bHLH DNA-binding domain and single amino acid substitutions such as R602M within the bHLH domain. Both types of alterations have been shown to drastically impact the ability of TCF12 to function as a transcription factor and interact with other bHLH proteins, eventually leading to mutant protein accumulation (Labreche et al., 2015). Patients harboring TCF12 mutations or LOH exhibited a shorter median OS. The frequency of these alterations was much higher in grade III AO as compared to grade II oligodendroglioma. This suggests that TCF12 alterations play a role in dictating an aggressive phenotype in AO. One analysis looking at the downstream effect of TCF12 alterations showed a downregulation of TCF21, EZH2, and BMI1 pathway and especially CDH1 (E-cadherin), which has been shown to be implicated in tumor characteristics and metastasis (Lee et al., 2012). Interestingly, it has been reported that TCF12 may have a haploinsufficient tumor suppressor role which increases the risk of developing AO in those patients harboring a TCF12 germline mutation.

### TERT Promoter Mutations

Human telomerase reverse transcriptase (TERT) mutations have been found to be present in 77% of grades II and III oligodendrogliomas and 82% of tumors with 1p19q co-deletion (Koelsche et al., 2013). Telomerase reverse transcriptase is a subunit of the enzyme telomerase that protects the overall integrity and length of telomeres. Telomerase normally functions to regenerate chromosomal ends (telomeres) thereby allowing DNA replication and mitosis. While usually unexpressed in mature cells, cancer cells make use of this enzyme to promote their survival and increase proliferation. TERT mutations in glioma are often found within the promoter region. This results in the opening of a binding site for the E26 transformation-specific transcription factors (Killela et al., 2013). TERT reactivation then takes place when GA-binding protein (GABP) transcription factor binds to the mutant TERT promoter (Dahlin et al., 2016).

In addition to being a surrogate for oligodendroglial lineage, TERT mutations seem to have some prognostic significance (Dahlin et al., 2016). Pekmezci et al. (2017) studied the status of both TERT and ATRX mutations along with their prognostic values in cohorts including grade II/III astrocytomas. The wildtype (WT) TERT group was associated with good prognosis only in IDH1/IDH2 WT (IDH-WT) grade II/III astrocytomas. However, in those groups with IDH mutations, including AO, TERT promoter mutation status was not a statistically significant prognostic factor (Dahlin et al., 2016). Thus, prognostic markers should be assessed while accounting for other genetic alterations.

Lately, IDH 1 and 2, which are known to generate nicotinamide adenine dinucleotide phosphate (NADPH), have been heavily observed. Their predictive value stems from their close relationship to human gliomas. Zou et al. (2013) was the first to conduct a meta-analysis on PFS and OS in gliomas based on IDH mutations. A better outcome was associated with IDH mutations and a combined HR estimate for OS and PFS was 0.33 (95% CI: 0.25–0.42) and 0.38 (95% CI: 0.21–0.68) for patients with gliomas harboring IDH mutation (Zou et al., 2013). A study by Kaminska et al. (2019) depicted how the mutant IDH1 (R132H) blocks cellular differentiation and contributes to antitumor immunity. Although a mutated IDH1 cannot generate NADPH since it has lost its normal catalytic activity, it gains the function of producing D-(R)-2-hydroxyglutarate. When the latter is overproduced in cancer cells, it inhibits histone and DNA methylases and interferes with cellular metabolism. The end result is DNA hypermethylation and thus the blockage of cellular differentiation (Kaminska et al., 2019).

### Other

The platelet-derived growth factor (PDGF) signaling system has been associated with the development and malignant progression of AO. Overexpression of PDGF system components, particularly the α subtype receptor (PDGFRα), was detected in Southern and Fluorescence *in situ* hybridization (FISH) analyses 4/41 AO. Although these tumors were not examined for correspondence between PDGFRα expression and PDGFRα gene amplification, application of the same methodology on studies involving EGFR indicate that a high level of protein expression is to be expected in the future (Smith et al., 2000b).

Finally, even though PTEN gene alterations have an unclear association with AO, their function in the control of cellular proliferation could explain their role in pathogenesis of AO. Sasaki et al. (2001) showed that 7/72 AO had PTEN gene alterations; 2 had homozygous DMBT1 deletions, but at least one reflected unmasking of a germline DMBT1 deletion. Moreover, no mutations were found in ERCC6 exon 2 and only two patients had a chemotherapeutic response, but with unexpected short survival times. Therefore, PTEN is a target of 10q loss, and PTEN alterations are associated with aggressive tumor phenotypes regardless of chemosensitivity (Sasaki et al., 2001).

### Clinical Trials and the CODEL Trial

There are 11 ongoing clinical trials recruiting patients with AO. NCT03971734 aims to determine the optimal dose of Regadenoson which alters the integrity of the Blood-Brain-Barrier in patients with high grade gliomas. Another phase 2 clinical trial (NCT04623931) is assessing chemotherapy and RT for the treatment of IDH wildtype gliomas or non-histological glioblastomas in approximately 40 patients. In an ongoing phase 3 study (NCT00887146), patients with AO or low-grade gliomas were split into two arms. Patients in arm A received RT with concomitant TMZ followed by adjuvant TMZ. Patients in arm B received RT first followed by PCV chemotherapy. Another clinical trial is a pediatric long-term follow-up and rollover phase 4 study (NCT03975829), whereby approximately 250 participants will be treated with dabrafenib and/or tametinib. NCT03434262 is a phase 1 study assessing the efficacy of different drugs on children and young adults. Each stratum has different combination treatments and targeted patient populations. While ribociclib is included as a treatment regimen across all strata, gemcitabine, trametinib, and sonidgib are included in strata A, B, and C, respectively. Elsewhere, another phase 1 study (NCT02644291) is assessing the use of mebendazole in recurrent/progressive pediatric brain tumors of 21 participants. Periclinal laboratory models have shown the efficacy of mebendazole against high grade gliomas and medulloblastomas.

NCT01849952 is another clinical trial that will evaluate the expression levels of microRNA-10b in patients with AO, although it will not involve any new therapeutic regimens. Investigators of this trial will be testing the *in vitro* sensitivity of individual primary tumors to anti-mir-10b treatment. Another currently ongoing phase I study (NCT04135807) is assessing the efficacy of an implantable microdevice in the brain before tumor resection is initiated. This microdevice will be used for 8 intratumor drugs: TMZ, Lomustine, Irinotecan, Carboplatin, Lapatinib, Osimertinib, Abenaciclib, and Everolimus. NCT04708548 is an ongoing European cross-sectional study that is looking at health-related quality of parameters and outcomes in survivors after being treated with surgery, chemotherapy and/or RT. The estimated completion date is August 2022.

NCT04541082 is another ongoing phase 1 study aiming to determine the maximum tolerated dose of the oral drug ONC206, a member of the imipridone class of anti-cancer small molecules which target G protein-coupled receptors. This trial aims to determine the maximum tolerated dose of ONC206. The efficacy and safety of other novel therapeutic drugs such as rQNestin34.5v.2 (an oncolytic viral vector) is also being assessed. As part of an ongoing phase 1 trial to treat recurrent malignant gliomas (NCT03152318), investigators hope that the rQNestin34.5v.2 drug will spread to a glioma cell, kill it, and then make a copy of itself and spread again. With approximately 108 participants included in this study, the estimated completion date is July 2022.

The CODEL study is a phase 3 study whereby 36 patients with newly diagnosed grade III oligodendrogliomas were randomized to receive RT alone (Arm A), RT with concomitant and adjuvant TMZ (Arm B) or TMZ alone (Arm C) (Jaeckle et al., 2021). At a median follow up of 7.5 years, around 80% (*n* = 10) patients in Arm C progressed vs. approximately 40% (*n* = 9) in the other arms. The HR was 3.12 with a 95% CI of 1.26–7.19 (*P* = 0.014) (Jaeckle et al., 2021). Even though there wasn’t any difference in OS, the PFS remained shorter for patients not receiving any RT; even after adjusting for IDH status and RT treatment status. The PFS HR was 3.33 with a 95% CI 1.31–8.45 (*P* = 0.011) while the OS HR was 2.78 with a 95% CI 0.58–13.22 (*P* = 0.20) (Jaeckle et al., 2021).

Lastly, it is worth noting that there are approximately 230 other clinical trials which involve oligodendrogliomas but are not actively recruiting patients.

## Conclusion

AO remains an understudied tumor with several unclear pathogenic pathways. Several genetic and protein alterations have been identified in AO. Some of these alterations have correlated with prognosis and response to treatment. Re-analysis of some trials prior to the 2016 WHO brain tumor classification has given further insight into some molecular pathways that were previously poorly defined or investigated. More studies, however, are needed to explore molecular pathways in oligodendroglioma and AO specifically after the 2016 classification.

## Author Contributions

MB drafted the manuscript and contributed to the discussion section. HA conceived the idea for the manuscript. Both authors have read and approved the final manuscript.

## Conflict of Interest

The authors declare that the research was conducted in the absence of any commercial or financial relationships that could be construed as a potential conflict of interest.

## Publisher’s Note

All claims expressed in this article are solely those of the authors and do not necessarily represent those of their affiliated organizations, or those of the publisher, the editors and the reviewers. Any product that may be evaluated in this article, or claim that may be made by its manufacturer, is not guaranteed or endorsed by the publisher.
